# Cost-effectiveness of single-pill and separate-pill administration of antihypertensive triple combination therapy: a population-based microsimulation study

**DOI:** 10.1186/s12889-024-19346-4

**Published:** 2024-07-06

**Authors:** Gabriella Morabito, Caterina Gregorio, Francesca Ieva, Giulia Barbati, Giuseppe Mancia, Giovanni Corrao, Federico Rea

**Affiliations:** 1 Department of Statistics and Quantitative Methods, Unit of Biostatistics, Epidemiology and Public Health, University of Milano-Bicocca, Via Bicocca Degli Arcimboldi, 8, Milan, 20126 Italy; 2 National Centre for Healthcare Research and Pharmacoepidemiology, University of Milano-Bicocca, Milan, Italy; 3 MOX - Modelling and Scientific Computing Laboratory, Department of Mathematics, Politecnico Di Milano, Milan, Italy; 4 Biostatistics Unit, Department of Medical Sciences, University of Trieste, Trieste, Italy; 5 Aging Research Center, Department of Neurobiology, Care Sciences and Society, Karolinska Institutet, Stockholm, Sweden; 6 Health Data Science Center, HDS, Human Technopole, Milan, Italy; 7 Emeritus Professor of Medicine, University of Milano-Bicocca, Milan, Italy

**Keywords:** Cost-effectiveness, Hypertension, Single-pill combination, Healthcare utilization database, Microsimulation, Real world

## Abstract

**Background:**

Single-pill combination (SPC) of three antihypertensive drugs has been shown to improve adherence to therapy compared with free combinations, but little is known about its long-term costs and health consequences. This study aimed to evaluate the lifetime cost-effectiveness profile of a three-drug SPC of an angiotensin-converting enzyme inhibitor, a calcium-channel blocker, and a diuretic *vs* the corresponding two-pill administration (a two-drug SPC plus a third drug separately) from the Italian payer perspective.

**Methods:**

A cost-effectiveness analysis was conducted using multi-state semi-Markov modeling and microsimulation. Using the healthcare utilization database of the Lombardy Region (Italy), 30,172 and 65,817 patients aged ≥ 40 years who initiated SPC and two-pill combination, respectively, between 2015 and 2018 were identified. The observation period extended from the date of the first drug dispensation until death, emigration, or December 31, 2019. Disease and cost models were parametrized using the study cohort, and a lifetime microsimulation was applied to project costs and life expectancy for the compared strategies, assigning each of them to each cohort member. Costs and life-years gained were discounted by 3%. Probabilistic sensitivity analysis with 1,000 samples was performed to address parameter uncertainty.

**Results:**

Compared with the two-pill combination, the SPC increased life expectancy by 0.86 years (95% confidence interval [CI] 0.61–1.14), with a mean cost differential of -€12 (95% CI -9,719–8,131), making it the dominant strategy (ICER = -14, 95% CI -€15,871–€7,113). The cost reduction associated with the SPC was primarily driven by savings in hospitalization costs, amounting to €1,850 (95% CI 17–7,813) and €2,027 (95% CI 19–8,603) for patients treated with the SPC and two-pill combination, respectively. Conversely, drug costs were higher for the SPC (€3,848, 95% CI 574–10,640 vs. €3,710, 95% CI 263–11,955). The cost-effectiveness profile did not significantly change according to age, sex, and clinical status.

**Conclusions:**

The SPC was projected to be cost-effective compared with the two-pill combination at almost all reasonable willingness-to-pay thresholds. As it is currently prescribed to only a few patients, the widespread use of this strategy could result in benefits for both patients and the healthcare system.

**Supplementary Information:**

The online version contains supplementary material available at 10.1186/s12889-024-19346-4.

## Background

Hypertension affects approximately 30% of the adult population worldwide and is a primary contributor to morbidity and mortality [1, 2]. Low adherence to long-term antihypertensive medication has been identified as a major factor in poor blood pressure (BP) control rates, substantially increasing the risk of death and hospitalization due to cardiovascular (CV) events [3]. Several studies have shown a direct correlation between the number of BP-lowering pills and poor adherence to medications, suggesting the potential benefit of using single-pill combination (SPC) therapy [4, 5]. The SPC allows patients to progress from one to two or three drugs while remaining on a simple treatment regimen with a single pill throughout, thereby enhancing the likelihood of adherence to therapy and achieving BP control. This approach has the potential to double BP control rates in treated patients, making SPC the preferred strategy for the initial two-drug combination treatment of hypertension and for three-drug combination therapy when required [6].

By definition, improvements in adherence to medications increase pharmacy spending but can lead to a reduction in direct and indirect costs by decreasing CV events. A systematic review indicated that high adherence to coronary artery disease treatment significantly improves outcomes prevention and can reduce expenditure from 10 to 18% [7], and a study based on administrative claims data in the United States showed a benefit–cost ratio from adherence of 10:1 in patients with hypertension [8]. If higher pharmacy costs are offset by reductions in the use of medical services, healthcare payers and policymakers would be motivated to adopt programs that promote compliance or remove barriers to adherence.

Regarding the SPC of three antihypertensive drugs, two retrospective population-based studies in Italy showed that patients treated with the SPC were more frequently adherent than those under multiple-pill administration and exhibited a lower risk of CV hospitalizations and reduction in healthcare costs [9, 10]. However, costs and outcomes were evaluated over a very short-time period, which does not provide a comprehensive understanding of the burden on the healthcare system, particularly considering the chronic nature of hypertension. A lifetime cost-effectiveness analysis of the three-drug SPC was conducted in the United States and revealed that the SPC was cost-effective compared with the corresponding free combination under a willingness-to-pay (WTP) threshold of $50,000 [11].

Therefore, the present study aimed to assess, in a real-world setting in Italy, the lifetime cost-effectiveness profile of single-pill and separate-pill administration of antihypertensive triple combination therapy from the payer perspective.

## Methods

The present study adhered to the reporting guideline of Consolidated Health Economic Evaluation Reporting Standards (CHEERS) [12].

### Setting

Data were extracted from the Healthcare Utilization Databases of Lombardy, a region in Italy representing nearly 16% (approximately 10 million) of its population. All Italian citizens have equal access to the health care services provided by the National Health Service (NHS), and in each Italian region, related data are recorded in an automated system of databases, which provides information on all health services free of charge, including drug dispensing in community and hospital pharmacies, (classified according to the Anatomical Therapeutic Chemical – ATC – classification system), hospitalizations (coded according to the International Classification of Diseases, 9th Revision, Clinical Modification [ICD-9-CM] classification system), and specialist visits and diagnostic examinations. The cost of each service provided to an NHS beneficiary and reimbursed to a health provider (i.e., direct healthcare cost for the Regional Health Authority) is also recorded routinely. These databases are linked by a unique individual identification code, which allows the tracking of the healthcare pathway of NHS beneficiaries. To ensure privacy, each identification code is anonymized, with the inverse process being only allowed to the Regional Health Authority upon request from judicial authorities. Additional details on the Healthcare Utilization Databases of Lombardy are available in previous publications [13, 14].

### Model overview

We developed a semi-Markov, continuous-time microsimulation model to estimate and compare the costs and life-years gained (LYs) with the SPC and two-pill combination over a lifetime horizon. This design allows to account for population heterogeneity and individual health history by simulating the outcomes on individual trajectories [15, 16]. The development of the model was performed in three steps: first, after defining the target population, the disease progression, costs, and effectiveness models were parametrized using available data. Second, these models were integrated into a single economic model, which was used to simulate the lifetime outcomes (costs and effects) in the target population. Finally, the simulated outcomes were used to perform the cost-effectiveness analysis.

### Study population

The target population consisted of residents of Lombardy aged ≥ 40 years. Among them, individuals who received a triple combination comprising an angiotensin-converting enzyme inhibitor (ACEI), a calcium-channel blocker (CCB), and a diuretic (D), either in a single pill (i.e., an SPC) or two pills (i.e., a two-drug SPC plus a third drug administered separately) between 2015 and 2018 were identified, and the date of the first dispensation was defined as the *index date*. Individuals who (i) were not beneficiaries of the NHS for at least 3 years before the *index date* and (ii) were already treated with ACEI/CCB/D combination in the year preceding the *index date* were excluded.

For model parametrization, cohort members were followed from the *index date* until the earliest occurrence of death, emigration, or December 31, 2019.

For each subject, demographic (i.e., sex and age at the *index date*) and clinical characteristics were assessed. To reconstruct patients’ clinical history, previous hospitalizations for CV disease and other causes, use of other drugs (such as statins and antidiabetic drugs), and clinical status were evaluated during the 3 years before the *index date*. Clinical status was determined using the Multisource Comorbidity Score, a prognostic score demonstrated to predict all-cause death among Italian individuals more accurately than other widely used scores [17]. Three categories of clinical profile were considered: good (0 ≤ score ≤ 4), intermediate (5 ≤ score ≤ 14), and poor (score ≥ 15). Additionally, the number of co-treatments and antihypertensive drugs dispensed during the year before the *index date* were considered to quantify pill-burden and identify antihypertensive drug strategy before switching to the three-drug combination. Moreover, the high-dimensional propensity score (HDPS) was calculated. The HDPS algorithm automatically identifies, from a vast amount of historical data, preexposure variables that proxy information for important unmeasured confounders, making it an established method for optimizing confounding capture and control with large healthcare databases [18]. The propensity for being prescribed the three-drug SPC was derived through a logistic regression model, incorporating the aforementioned baseline characteristics plus the 200 most predictive covariates selected by the HDPS algorithm. To consider the possible impact of calendar time on the probability of being prescribed the three-drug SPC, the year of the first ACEI/CCB/D combination dispensation was included in the HDPS calculation.

Standardized mean differences were employed to assess differences between groups [19], with differences < 0.10 considered negligible.

### Disease model

**Fig. 1:**
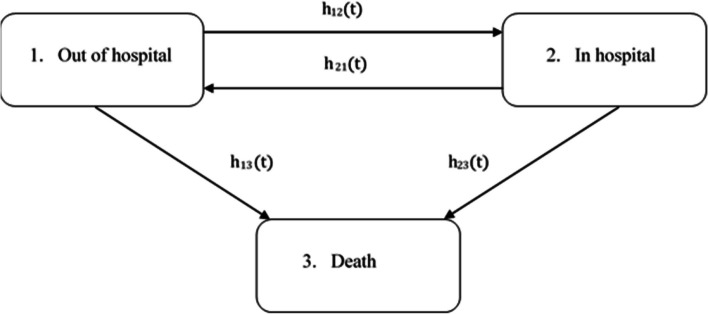
The three-state model of disease progression. ${h}_{rs}\left(t\right)$ represents the hazard for transitioning from the state *r* to the state *s* at time *t*

To reconstruct the disease progression for each patient of the selected cohort conditional on the treatment used, an individual-level state-transition model was employed, and all hospitalizations for major CV diseases (i.e., heart failure, stroke, and myocardial infarction) until death were assessed. As shown in Fig. 1, three mutually exclusive health states were considered: *Out of hospital*, *In hospital*, and *Death*. To estimate the transition intensities, a flexible parametric survival model with four knots was employed to accommodate different shapes for hazard [20]. The Markov assumption was relaxed by employing a *clock-reset* time scale, i.e., by resetting time to zero whenever a patient enters a new state. This framework allows the hazard to change at each transition, introducing dependence on the past through time spent in the present state [21]. The model was adjusted for the aforementioned covariates, i.e., age, sex, clinical status, and HDPS.

### Costs and effectiveness models

Direct healthcare costs included hospitalization for major CV diseases, antihypertensive drugs, and outpatient services for CV care (such as specialist visits, laboratory examinations, and imaging). For each cost category, the mean and standard error were estimated. In particular, the average daily cost of outpatient services and hospitalizations was considered for the *Out of hospital* and *In hospital* states, respectively. The average daily drug cost for the two treatment strategies (i.e., SPC vs two-pill combination) was estimated from the data using a generalized linear model adjusted for age, sex, clinical status, and HDPS. All costs were expressed in euros (€).

The effectiveness of each strategy was assessed through LYs.

### Simulation

The parametrized models described in the previous sections were integrated into an economic model to simulate individuals’ trajectories. The selected cohort served as input data, assigning each strategy to each patient. Additionally, costs and LYs were discounted at an annual rate of 3% [22].

### Decision analysis

The simulated costs and LYs were used to calculate the incremental cost-effectiveness ratio (ICER). The SPC was considered cost-effective if the ICER was below a WTP threshold of €23,000. This value was derived through an algorithm based on Italian per-capita health expenditures and life expectancy [23]. To evaluate whether cost-effectiveness outcomes could be influenced by patients’ characteristics, the analysis was repeated separately for subgroups according to sex, age, and clinical status.

### Sensitivity analyses

To account for parameter uncertainty, we conducted a probabilistic sensitivity analysis (PSA) by simultaneously sampling 1,000 model inputs from defined probabilistic distributions [24]. As costs were right skewed, they were assumed to follow a Gamma distribution, with shape and scale parameters derived through the method of the moment from the estimated mean and standard error. For transition hazards, the asymptotic normal distribution of the maximum likelihood estimate was used. Subsequently, marginal and conditional (for each subgroup) ICERs were calculated by averaging the total LYs and costs across each patient and PSA sample. The 95% confidence intervals (CIs) were obtained from the distribution of the PSA samples, with the 2.5th and 97.5th percentiles serving as the lower and upper bound, respectively.

Additional sensitivity analyses were performed to assess the robustness of the results. First, the analysis was repeated without discounting. Second, the cost model was reparametrized by considering costs related to all outpatient services, rather than just those for CV care, to evaluate whether increased event-free survival could impact the cost-effectiveness profile through a more intense use of healthcare services. Third, to assess the adequacy of the microsimulation model in approximating observed data, individuals’ trajectories were simulated over a short-time horizon equal to the maximum observation period of the cohort. To obtain the observed incremental LYs, restricted mean survival time was estimated for the two groups through pseudovalue regression [25], while incremental costs were calculated by fitting a linear model. Estimates were adjusted for the previously mentioned covariates.

### Software

The Statistical Analysis System Software (version 9.4; SAS Institute, Cary, NC) and R Statistical Software (version 3.6.1; R Foundation for Statistical Computing, Vienna, Austria) were used for the analyses. For all hypotheses tested, two-tailed P-values < 0.05 were considered significant.

## Results

### Study cohort

Among patients receiving the ACEI/CCB/D combination from 2015 through 2018, 30,172 and 65,817 incident users of the three-drug SPC and two-pill combination, respectively, met the inclusion and exclusion criteria. The cohort members accumulated 244,952 person-years of observation, with a median follow-up of 31 months. Maximum follow-up time was of 52 months.

The baseline characteristics of the cohort members are shown in Supplementary Table S1, Additional file 1. Compared with patients who were prescribed the three-drug two-pill combination, those treated with a three-drug SPC were younger, more frequently male, and had a slightly better clinical profile. Moreover, the two groups had received different antihypertensive therapies in the year before the *index date*.

### Parametrization

Table 1 shows the results of the parametrization from the disease and cost models. Compared with the two-pill combination, the SPC exhibited a significant protective effect on CV hospitalization (hazard ratio = 0.91, 95% CI 0.85–0.96) and all-cause death (0.79, 0.73–0.85) and slightly increased the probability of being discharged after a hospitalization (1.09, 1.02–1.16). However, no significant effect of the strategy on the hospitalization-related mortality risk was observed. The average daily drug cost was slightly higher for the SPC.

**Table 1:** Results from the disease and costs models

Parameter	Value (95% CI/SD)
Effect of SPC vs. two-pill combination on transition probabilities (hazard ratios)	
* Out of hospitalIn Hospital*	0.91 (0.85–0.96)
* Out of hospitalDeath*	0.79 (0.73–0.85)
* In hospitalOut of hospital*	1.09 (1.02–1.16)
* In hospitalDeath*	0.88 (0.68–1.14)
Average daily costs (€)	
Drugs	
SPC	0.54 (0.35)
Two-pill combination	0.52 (0.43)
CV Hospitalizations	602.01 (684.00)
CV Outpatient services	0.15 (0.33)

*CI* confidence interval, *SD* standard deviation, *SPC* single-pill combination, *CV* cardiovascular

### Base case analysis

Simulated total CV costs were €6,718 (95% CI 1,478–15,914) and €6,729 (95% CI 1,196–17,913) for the SPC and two-pill combination, respectively (Table 2). The reduction in expenditure for the SPC was driven by savings in hospitalization costs, which amounted to €1,850 (95% CI 17–7,813) and €2,027 (95% CI 19–8,603) for patients treated with the SPC and two-pill combination, respectively. Conversely, drug costs were higher for the SPC (€3,848, 95% CI 574–10,640 vs. €3,710, 95% CI 263–11,955). Patients taking the three antihypertensive drugs as the SPC and two-pill combination had a mean survival of 19.54 (95% CI 19.06–19.97) and 18.68 (95% CI 18.20–19.12) years, respectively.

**Table 2:** Simulated CV costs (euros), stratified by category, and LYs for each treatment strategy

	**SPC**	**Two-pill combination**
Costs (95% CI)		
Overall	6718 (1478–15914)	6729 (1196–17913)
Drugs	3848 (574–10640)	3710 (263–11955)
Hospitalizations	1850 (17–7813)	2027 (19–8603)
Outpatient services	1020 (0–6434)	992 (0–6072)
LYs (95% CI)	19.54 (19.06–19.97)	18.68 (18.20–19.12)

*CI* confidence interval, *CV* cardiovascular, *SPC* single-pill combination, *LYs* life-years gained

**Fig. 2:**
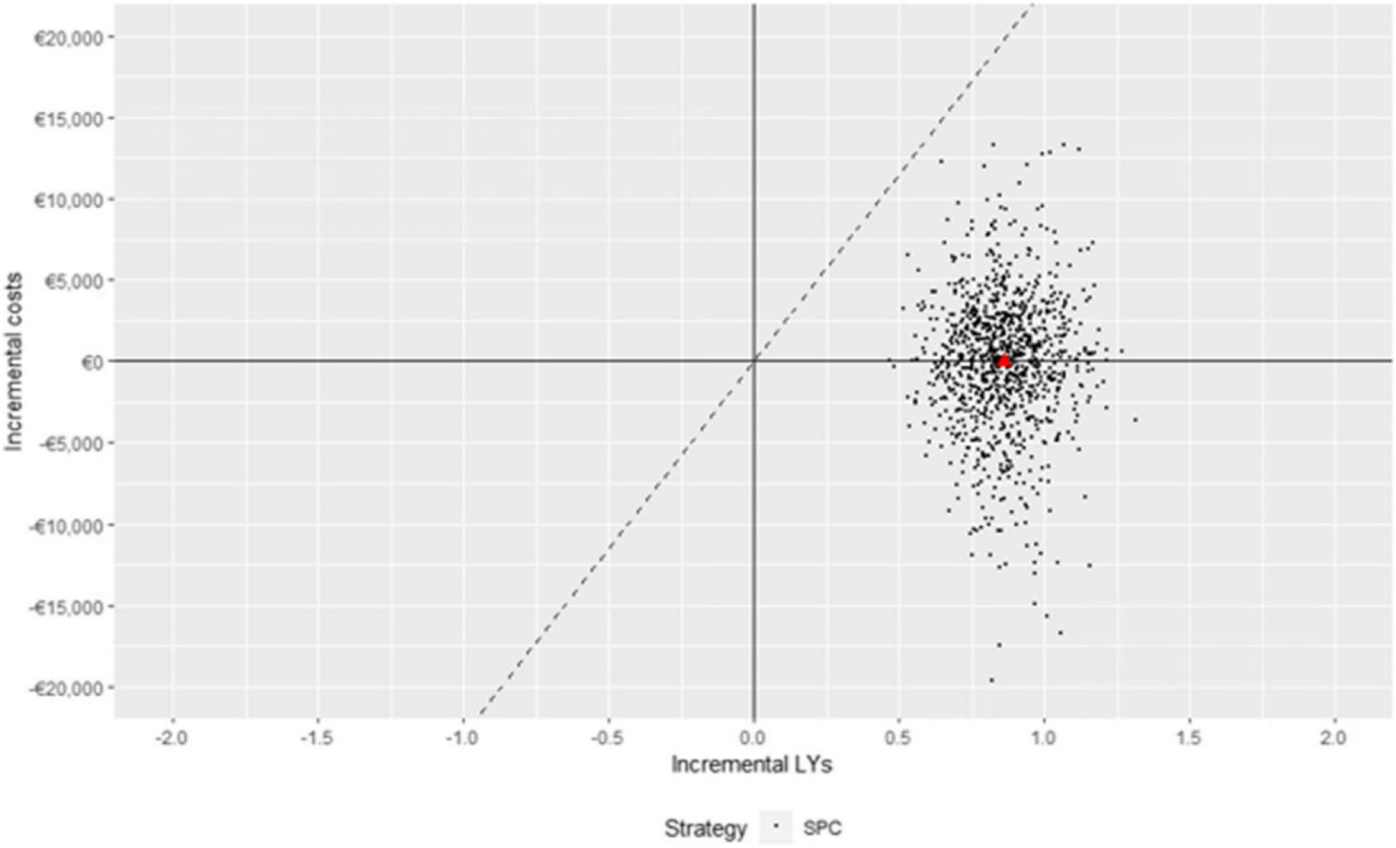
Cost-effectiveness plane. The plot demonstrates both the uncertainty and magnitude of the estimates. Each point on the plot is from a particular random draw from the PSA. Red triangle represents the mean ICER across the PSA samples. The dotted line is the reference WTP threshold (€23,000). Abbreviations: ICER, incremental cost-effectiveness ratio; PSA, probabilistic sensitivity analysis; SPC, single-pill combination; WTP, willingness-to-pay

The cost-effectiveness results are presented in Fig. 2. On average, compared with patients under the two-pill combination, patients treated with the SPC gained 0.86 (95% CI 0.61–1.14) LYs, with a cost differential of -€12 (95% CI -9,719–8,131), resulting in an average saving of €14 per LY (ICER of -14, 95% CI -15,871–7,113). The cost-effectiveness profile did not significantly change according to age, sex, and clinical profile (Table 3).

**Table 3:** Cost-effectiveness results according to subgroups

Strata	Incremental costs (95% CI)	Incremental LYs (95% CI)	ICER (95% CI)
Sex			
Male	2 (-9810–8251)	0.83 (0.58–1.09)	1 (-15522–6993)
Female	-23 (-9656–8033)	0.90 (0.63–1.18)	–28 (-16515–7337)
Age (years)			
40–64	-76 (-13006–10805)	0.48 (0.32–0.66)	-156 (-40984–16311)
65–80	-17 (-8703–7204)	0.94 (0.66–1.25)	-18 (-13229–5779)
> 80	363 (-6631–5769)	1.32 (0.96–1.76)	83 (-6934–3280)
Clinical profile^a^			
Good	-36 (-10843–9044)	0.70 (0.48–0.94)	-51 (-22386–9628)
Intermediate	11 (-7812–6524)	1.19 (0.85–1.57)	8 (-9208–4159)
Poor	215 (-4310–4027)	1.29 (0.80–1.66)	185 (-5352–2416)

*CI* confidence interval, *LYs* life-years gained, *ICER* incremental cost-effectiveness ratio

^a^Three categories were considered for the clinical profile according to the Multisource Comorbidity Score: good (0 ≤ score ≤ 4), intermediate (5 ≤ score ≤ 14), and poor (score ≥ 15)

### Sensitivity analyses

According to PSA, the SPC was dominant (i.e., both more effective and cost-saving) for 46% of samples and had a probability of being the most cost-effective strategy close to 100% for almost all reasonable values of the WTP threshold (Supplementary Figure S1, Additional file 1).

Results did not significantly change without discounting costs and LYs (ICER = 71, 95% CI -13,824–6,260) and by reparametrizing costs (ICER = 505, 95% CI -15,233–7,910) (Supplementary Figures S2–S3, Additional file 1). The short-time (52 months) microsimulation provided comparable results to those observed in the study cohort, with the SPC proving to be dominant according to both approaches (Supplementary Table S2, Additional file 1).

## Discussion

This study provides the first lifetime cost-effectiveness analysis of the SPC of three BP-lowering drugs compared with the corresponding two-pill combination within the Italian healthcare system, using a flexible decision model based on real-world data.

The present study reveals three primary findings. First, the SPC was dominant, indicating that patients prescribed the three-drug SPC experienced more life-years than those prescribed the two-pill combination (positive differential effectiveness), coupled with cost savings (negative healthcare costs differential). While our data suggest an increase in drug costs associated with the SPC, a reduction in hospitalization costs contributes to an overall decrease in total health expenditure. Second, the SPC proved to be cost-effective at almost all reasonable WTP thresholds (see below). Third, the cost-effectiveness profile remained consistent across age, sex, and clinical status.

Evaluating cost-effectiveness profiles in Italy is challenging due to the absence of a widely accepted reference threshold in the literature. The WTP threshold per year of life gained, commonly adopted by Western countries, typically ranges from €30,000 to €95,000 [26], while Italy’s GDP per capita is approximately €35,000 [27]. Recently, an algorithm was developed to estimate the most suitable WTP threshold based on national per-capita health expenditures, life expectancy, and health outcomes considered in the analysis [22]. Following this approach, we selected a threshold of €23,000, which is also the most conservative value compared with those usually employed. Our study demonstrates that the additional healthcare cost per year gained that the payer should bear with the SPC compared with the two-pill combination was well below this threshold. This was the case regardless of the subgroups and scenarios considered, as well as according to PSA. Even when setting a lower WTP threshold, such as €10,000, the probability of the SPC being cost-effective was approximately 98%.

There are noteworthy findings beyond the primary results. First, the SPC was used by only 31% of patients initiating the ACEI/CCB/D combination, and this strategy was less adopted by women, frail individuals, and the very elderly. Despite being the most commonly used two-drug combination strategy in Lombardy [28], the same does not hold true for the three-drug combination. Because the use of the SPC could translate into benefits for patients, there is much room for improvement. Second, compared with the two-pill combination, the SPC was associated with a 9% lower risk of CV hospitalization and a 21% reduction in mortality risk. Considering that the perindopril/amlodipine/indapamide SPC demonstrated similar efficacy and tolerability to the same combination given in two separate pills in randomized controlled trials [29], the observed benefits of the SPC likely stem from improved drug compliance. Indeed, a lower pill burden has been shown to be associated with higher adherence (+ 138%) in a previous investigation of our group [9], whose inverse relationship with CV risk is widely known [3, 30, 31].

Our findings align with those reported in a recent Italian population-based study, demonstrating that compared with the corresponding two-pill combination, the three-drug SPC led to a 13% reduction in CV hospitalization risk through improved adherence and was associated with lower healthcare costs during the first year of treatment [9]. Similar results were obtained in another Italian observational study [10]. Our results complement these evaluations by providing a long-term cost-effectiveness analysis with a decision model-based approach. Another study, conducted on patients enrolled in a Medicare advantage plan in the United States, assessed the cost-effectiveness profile of three BP-lowering drugs assumed as an SPC and multiple-pill regimen [11]. The authors constructed a 5-year cycle Markov model that incorporated treatment adherence, finding that the SPC was cost-effective at a WTP threshold of $50,000. However, when comparing these results, one must keep in mind that (i) economic and health context is quite dissimilar between the two nations, (ii) the study used quality-adjusted life-years instead of LYs, and (iii) the modeling frameworks were different.

This study has several strengths. First, since Italy’s NHS encompasses virtually all citizens, our analyses were conducted on a very large and unselected population. This translates into a high degree of generalizability of our findings, even to frail patients who are generally excluded from trials. Second, all health services and related costs supplied to patients are meticulously recorded in our databases, known for their accuracy as health providers are required to report services in detail to obtain reimbursement, with legal consequences for incorrect reports [32]. Therefore, the use of healthcare utilization databases is particularly suitable for economic evaluations in real-world clinical practice. Third, adopting a decision modeling framework allowed for a cost-effectiveness analysis in a lifetime horizon, which is crucial for comprehensively assessing the impact on the healthcare system of treatments for chronic conditions such as hypertension. Moreover, the flexible parametric multi-state model employed, combined with microsimulation, allowed to overcome the limitations of the Markov assumption. Indeed, although Markov decision models are standard in cost-effectiveness analyses for chronic illnesses, their unsuitability in modeling healthcare paths has already been reported in the literature [16, 33]. Additionally, this design, involving estimating individuals’ trajectories, enabled the examination of cost-effectiveness profiles for groups of patients with different characteristics. This may prove useful in identifying subgroups of patients who would benefit most from treatment. Finally, our results were confirmed using several sensitivity analyses. In particular, the short-term simulation provided similar results to those observed in the cohort during the follow-up, suggesting that the economic model was correctly specified.

This study also has some limitations. First, the model assumes that patients remained on the same treatment strategy throughout the duration of the simulation. Although treatment switching could impact cost-effectiveness results, the low switch rates (1.2% and 2.1% of patients switched from the two-pill combination to the SPC and vice versa, respectively) during the follow-up suggested that such switches are unlikely to have a significant impact on the cost-effectiveness profile. Second, owing to the lack of data on quality of life, we could not measure endpoints such as quality-adjusted life-years, consequently the cost-utility profile. Third, because antihypertensive drugs are also prescribed for coronary heart disease and heart failure, our results may be affected by an unbalanced distribution of CV diseases between the two groups. However, antihypertensive treatment constitutes the predominant use of ACEIs, CCBs, and diuretics in Italy [34], especially when these drugs are used in combination. Fourth, the exclusion of prevalent users limits the generalizability of our findings. Fifth, only the perindopril/amlodipine/indapamide SPC was available in the Italian market during the study follow-up (2015–2019) as a brand-name drug. Consequently, the cost-effectiveness profile might change with the introduction of generic formulations. However, this scenario would lead to lower pharmacy spending for the SPC, making the cost-effectiveness profile even more favorable. Sixth, it should be remembered that the lifetime simulation was parameterized over a relatively short observation period, hence, by definition, it cannot take into account unobserved aging-related phenomena that may affect the final simulated cost and life expectancy. However, this should not influence the cost-effectiveness profile, as aging-related factors likely affect both groups equally. Finally, due to the observational nature of the study and the lack of clinical information in our databases (e.g., BP levels), the results might be influenced by confounding. While adjusting for the HDPS aims to minimize bias, potential unmeasured confounding cannot be entirely ruled out.

## Conclusions

In conclusion, the three-drug SPC was projected to be cost-effective compared with the two-pill combination. This study strongly advocates for the use of the SPC when three drugs are needed to achieve BP control. Since it is currently prescribed to a few patients, widespread use of this strategy could translate into benefits for both patients and the healthcare system.

### Supplementary Information

Supplementary Material 1.

## Data Availability

The data that support the findings of this study are available from Lombardy Region but restrictions apply to the availability of these data, which were used under license for the current study, and so are not publicly available. Data are however available from the corresponding author upon reasonable request and with permission of Lombardy Region.

## Abbreviations

ACEI: Angiotensin-converting enzyme inhibitor
BP: Blood pressure
CCB: Calcium-channel blocker
CI: Confidence interval
CV: Cardiovascular
D: Diuretic
ICER: Incremental cost-effectiveness ratio
HDPS: High-dimensional propensity score
LYs: Life-years gained
NHS: National Health Service
PSA: Probabilistic sensitivity analysis
SPC: Single-pill combination
WTP: Willingness-to-pay
