# Supplementary material for: Cost-effectiveness of single-pill and separate-pill administration of antihypertensive triple combination therapy: a population-based microsimulation study
Source: BMC Public Health. 2024 Jul 6;24:1808. doi: 10.1186/s12889-024-19346-4 (PMC11227134; doi:10.1186/s12889-024-19346-4)
Supplement: Supplementary file 1 — Supplementary Material 1. [file 12889_2024_19346_MOESM1_ESM.docx]

**Additional file 1**

**Cost-effectiveness of single-pill and separate-pill administration of antihypertensive triple combination therapy: a population-based microsimulation study**

Gabriella Morabito^1,2^, Caterina Gregorio^3,4,5^, Francesca Ieva^2,3,6^, Giulia Barbati^2,4^, Giuseppe Mancia^7^, Giovanni Corrao^1,2^, Federico Rea^1,2^

**Supplementary Table S1.** Baseline characteristics and mortality rates of patients prescribed the three-drug single-pill combination (SPC) and the two-pill combination.

|  | **Patients on SPC (N=30,172)** | **Patients on two-pill combination (N=65,817)** | **Standardized mean differences** |
| --- | --- | --- | --- |
| Men | 15,815 (52.4 %) | 30,119 (45.8 %) | -0.133 |
| Age (years) |  |  | -0.264 |
| 40–64 | 12,019 (39.8 %) | 19,776 (30.1 %) |  |
| 65–80 | 13,951 (46.2 %) | 31,740 (48.2 %) |  |
| >80 | 4,202 (13.9 %) | 14,301 (21.7 %) |  |
| Previous BP-lowering therapy * |  |  | 0.363 |
| No antihypertensive drugs | 2,487 (8.2 %) | 1,848 (2.8 %) |  |
| Monotherapy | 3,006 (10.0 %) | 3,830 (5.8 %) |  |
| Two drugs | 10,125 (33.6 %) | 32,523 (49.4 %) |  |
| Three or more drugs | 14,554 (48.2 %) | 27,616 (42.0 %) |  |
| Previous hospitalizations ^#^ |  |  |  |
| Stroke | 819 (2.7 %) | 2,017 (3.1 %) | -0.021 |
| Heart failure | 355 (1.1 %) | 1,445 (2.2 %) | -0.079 |
| Myocardial infarction | 423 (1.4 %) | 1,243 (1.9 %) | 0.038 |
| Diabetes | 915 (3.0 %) | 2,101 (3.2 %) | 0.009 |
| Kidney disease | 330 (1.1 %) | 1,135 (1.7 %) | -0.054 |
| Clinical profile ^§ #^ |  |  | 0.110 |
| Good | 21,853 (72.4 %) | 43,925 (66.7 %) |  |
| Intermediate | 7,223 (23.9 %) | 18,522 (28.1 %) |  |
| Poor | 1,096 (3.6 %) | 3,370 (5.1 %) |  |
| Number of co-treatments * |  |  | 0.062 |
| 0–4 | 17,812 (59.0 %) | 37,031 (56.3 %) |  |
| 5–9 | 9,749 (32.3 %) | 22,269 (33.8 %) |  |
| ≥10 | 2,611 (8.7 %) | 6,517 (9.9 %) |  |
| Mortality rate (1,000 person-years) | 15.48 | 27.49 |  |

Abbreviations: SD, standard deviation; BP, blood pressure.

^§^ Three categories were considered for the clinical profile according to the Multisource Comorbidity Score: good (0 ≤ score ≤ 4), intermediate (5 ≤ score ≤ 14), and poor (score ≥ 15).

* In the year before the *index date*.

^#^ In the 3-year period before the *index date*.

**Supplementary Table S2.** Comparison of outcomes obtained from the short-time microsimulation model with those observed in the cohort.

| **Outcome** | **Microsimulation** | **Observed** |
| --- | --- | --- |
| Incremental LYs | 0.04 | 0.04 |
| Incremental costs (€) | -56 | -32 |
| ICER | Dominant | Dominant |

**Supplementary Figure S1.** Cost-effectiveness acceptability curves of single-pill combination (SPC) and two-pill combination.


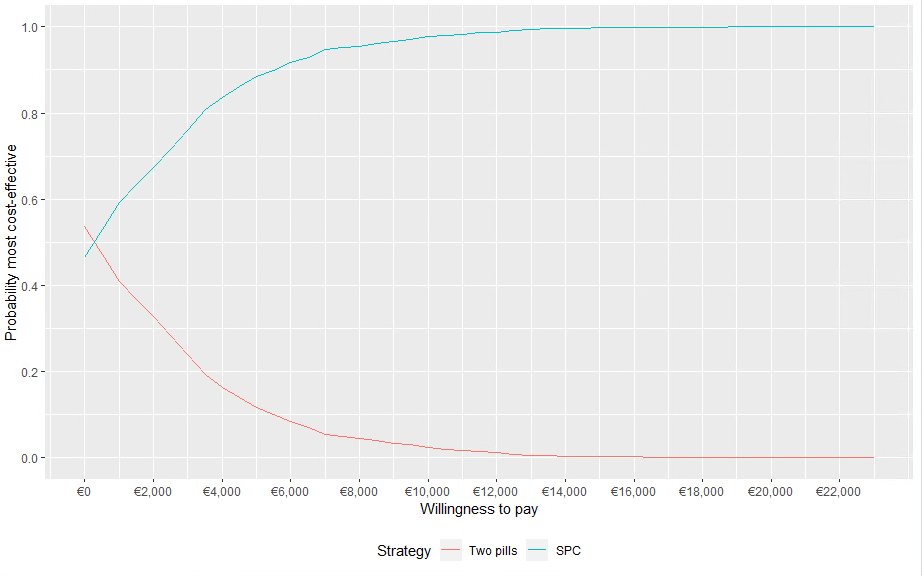


**Supplementary Figure S2.** Cost-effectiveness plane representing the incremental effectiveness and the incremental cost of the single-pill combination (SPC) compared to the two-pill combination without discounting.


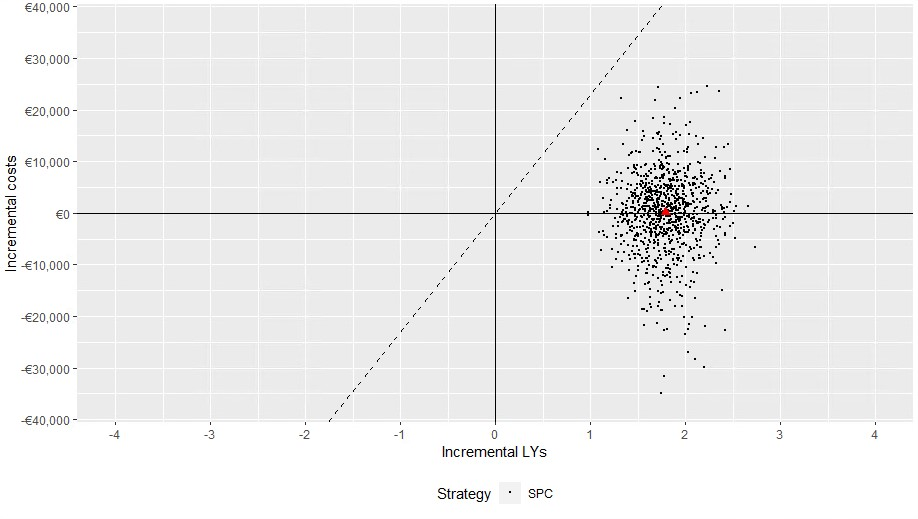


Each point on the plot is from a particular random draw from the PSA. Red triangle represents the mean ICER across the PSA samples. The dotted line is the reference WTP threshold (€23,000).

**Supplementary Figure S3.** Cost-effectiveness plane representing the incremental effectiveness and the incremental cost of the single-pill combination (SPC) compared to the two-pill combination, considering costs for all outpatient services.


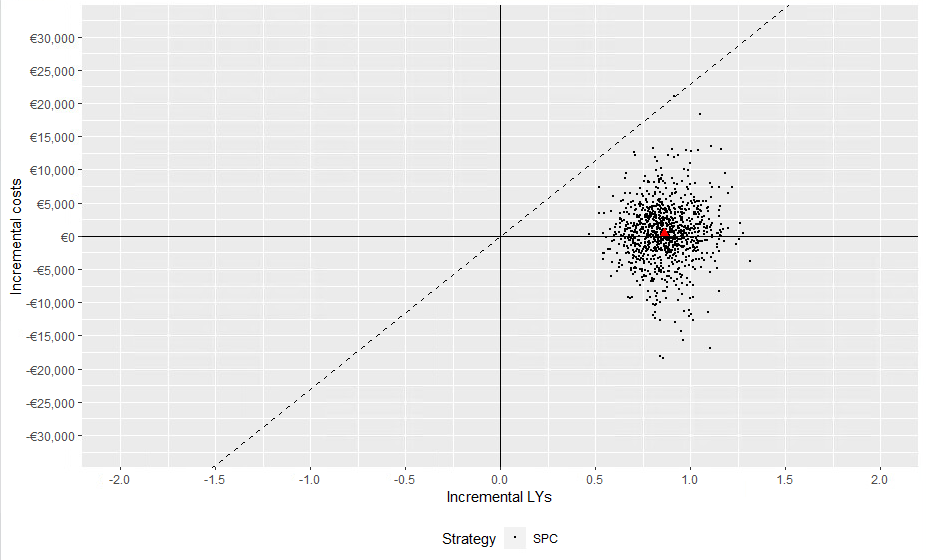


Each point on the plot is from a particular random draw from the PSA. Red triangle represents the mean ICER across the PSA samples. The dotted line is the reference WTP threshold (€23,000).
